# Preface

**DOI:** 10.2188/jea.15.S1

**Published:** 2005-05-16

**Authors:** Takesumi Yoshimura, Yutaka Inaba, Yoshinori Ito, Shuji Hashimoto, Akiko Tamakoshi, Yoshiyuki Watanabe

**Affiliations:** 1 Fukuoka Institute of Health and Environmental Sciences, Former affiliation: Department of Clinical Epidemiology, Institute of Industrial Ecological Sciences, University of Occupational and Environmental Health, Japan.; 2 Department of Epidemiology and Environmental Health, Juntendo University School of Medicine.; 3 Department of Public Health, Fujita Health University School of Health Sciences.; 4 Department of Hygiene, Fujita Health University School of Medicine.; 5 Department of Preventive Medicine/Biostatistics and Medical Decision Making, Nagoya University Graduate School of Medicine.; 6 Department of Epidemiology for Community Health and Medicine, Kyoto Prefectural University of Medicine Graduate School of Medical Science.

The Japan Collaborative Cohort Study (JACC Study) for Evaluation of Cancer Risk sponsored by the Ministry of Education, Science, Sports and Culture of Japan (Monbusho) was initiated in 1986 by two distinguished scientists -- Dr. Kunio Aoki, Professor Emeritus, Nagoya University School of Medicine, and Dr. Haruo Sugano, the former Director of the Cancer Institute, Tokyo -- in collaboration with 36 active epidemiologists at 24 different institutions.^1^^-^^3^

Approximately 127,500 healthy inhabitants from 45 areas throughout Japan who responded to the study questionnaire between 1988 and 1990, were enrolled into the study. Among this group, a total of 110,792 individuals (46,465 men and 64,327 women) aged 40-79 years were followed up for a period of at least 10 years. Descriptions for the initial stage of the JACC Study have been published elsewhere.^1^^-^^3^

Sixteen years have now passed since the JACC Study was first established with the support of Monbusho (Table). Throughout this period, especially during the past 5 years, members of the JACC Study Group have published a lot of original papers in various medical journals. Each paper has focussed on a specific scientific aspect of the research, in order to test specific hypothesis derived from the JACC Study and latest findings of cancer epidemiology. However, each original paper, mainly hypothesis testing type, might not help us to understand background, characteristics and nature of the JACC Study. Therefore, we realized that descriptive information and validation study for main study items of the JACC Study should be published for better and clear understanding of the JACC Study. This is the reason why the members of the JACC Study Group (chairperson: Akiko Tamakoshi) decided to publish related articles by the project. Yutaka Inaba, Yoshinori Ito, Shuji Hashimoto, Akiko Tamakoshi, Yoshiyuki Watanabe and Takesumi Yoshimura were nominated as members of the Report Publishing Committee (chairman: Takesumi Yoshimura), which was established with the support of a Monbusho grant in 2001. On the recommendation of the Committee, the articles were collected in the supplement of the Journal of Epidemiology with the committed assistance of the Japan Epidemiological Association.

**Table.**
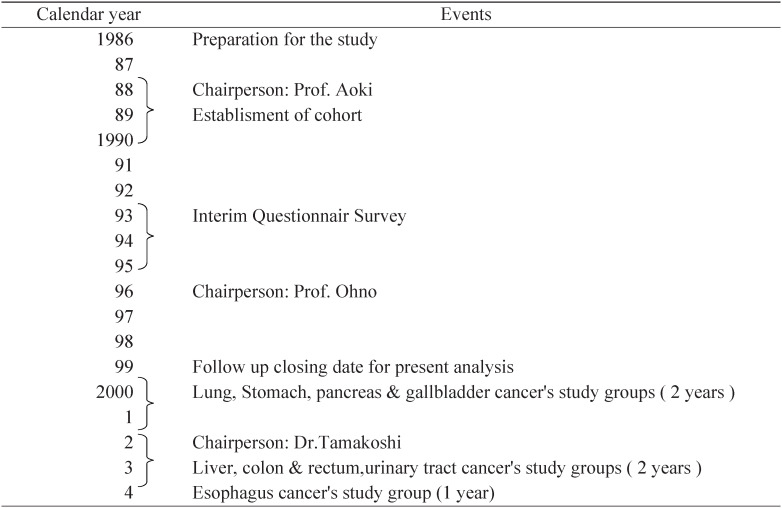
History of the JACC Study

The aims of the present supplement are: to present a summary of the data derived from the JACC Study; to clarify the details of the project; and to publish validity studies for each variable investigated, including major lifestyle factors such as smoking, drinking, dietary habits, physical exercise and serum chemistry.

The authors anticipate that this supplement will be a valuable resource for better understanding of the JACC Study.

## MEMBER LIST OF THE JACC STUDY GROUP

The present investigators involved, with the co-authorship of this paper, in the JACC Study and their affiliations are as follows: Dr. Akiko Tamakoshi (present chairperson of the study group), Nagoya University Graduate School of Medicine; Dr. Mitsuru Mori, Sapporo Medical University School of Medicine; Dr. Yutaka Motohashi, Akita University School of Medicine; Dr. Ichiro Tsuji, Tohoku University Graduate School of Medicine; Dr. Yosikazu Nakamura, Jichi Medical School; Dr. Hiroyasu Iso, Institute of Community Medicine, University of Tsukuba; Dr. Haruo Mikami, Chiba Cancer Center; Dr. Yutaka Inaba, Juntendo University School of Medicine; Dr. Yoshiharu Hoshiyama, University of Human Arts and Sciences; Dr. Hiroshi Suzuki, Niigata University School of Medicine; Dr. Hiroyuki Shimizu, Gifu University School of Medicine; Dr. Hideaki Toyoshima, Nagoya University Graduate School of Medicine; Dr. Kenji Wakai, Aichi Cancer Center Research Institute; Dr. Shinkan Tokudome, Nagoya City University Graduate School of Medical Sciences; Dr. Yoshinori Ito, Fujita Health University School of Health Sciences; Dr. Shuji Hashimoto, Fujita Health University School of Medicine; Dr. Shogo Kikuchi, Aichi Medical University School of Medicine; Dr. Akio Koizumi, Graduate School of Medicine and Faculty of Medicine, Kyoto University; Dr. Takashi Kawamura, Kyoto University Center for Student Health; Dr. Yoshiyuki Watanabe, Kyoto Prefectural University of Medicine Graduate School of Medical Science; Dr. Tsuneharu Miki, Graduate School of Medical Science, Kyoto Prefectural University of Medicine; Dr. Chigusa Date, Faculty of Human Environmental Sciences, Mukogawa Women’s University ; Dr. Kiyomi Sakata, Wakayama Medical University; Dr. Takayuki Nose, Tottori University Faculty of Medicine; Dr. Norihiko Hayakawa, Research Institute for Radiation Biology and Medicine, Hiroshima University; Dr. Takesumi Yoshimura, Fukuoka Institute of Health and Environmental Sciences; Dr. Akira Shibata, Kurume University School of Medicine; Dr. Naoyuki Okamoto, Kanagawa Cancer Center; Dr. Hideo Shio, Moriyama Municipal Hospital; Dr. Yoshiyuki Ohno, Asahi Rosai Hospital; Dr. Tomoyuki Kitagawa, Cancer Institute of the Japanese Foundation for Cancer Research; Dr. Toshio Kuroki, Gifu University; and Dr. Kazuo Tajima, Aichi Cancer Center Research Institute.
